# East Tennessee

**Published:** 1874-04-15

**Authors:** F. K. Bailey

**Affiliations:** Knoxville, Tenn.


### EAST TENNESSEE.—ITS CLIMATE, PREVAILING DISEASES, ETC.

BY F. K. BAILEY, M.D., KNOXVILLE, TENN.

EDITORS OF EXAMINER: I often receive letters from medical men and others, inquiring in regard to East Tennessee, its climate, prevailing diseases, etc., and also whether I would advise invalids to come here.

In the first place, I can say that, after having spent seven winters in Knoxville, the climate from November to April is decidedly mild. *Once* during the time (winter of 1872–73) the mercury went below zero. In our coldest days it is rare to find it below 10° above. During the same winter twenty-two inches of snow fell. With that exception, it has seldom aggregated more than from six to ten inches during the season. Rain is frequent in winter, taking the place of snow at points further north. It is rare for the ground to freeze more than three or four inches. Dampness is perhaps the prevailing condition of the atmosphere, although we sometimes have fifteen days in a month without rain. It is never so cold as to require the face to be muffled; and frozen ears or cheeks are unheard of. Still, as cold is a relative term, there is sometimes much discomfort felt, unless a person is warmly clothed. With a warm suit of underclothing, it is very easy to keep warm, even while riding in the coldest days. Perhaps one-half of the people never wear an overcoat. With proper care in the construction of houses, the people can be comfortable with an amount of fuel which, in the North, would only suffice in March or April weather. During the winter just passed, there has been but little cold weather. But four inches of snow have fallen; and our people must content themselves with ice less than two inches in thickness. There were but three or four days in which even that could be secured.

In regard to this region being a place of winter-residence for invalids, I have no doubt of its advantages. The few who come from year to year are generally benefited, and speak with delight of the balmy air on our coldest days, in contrast with the piercing blasts of northern latitudes. This is also a desirable point for those who have spent the winter in Florida ; since they can stop here in the spring months, before going to their northern homes.

There is no question as to asthmatics being benefited by a residence in this climate. Persons afflicted with catarrhal affections generally feel relieved at once. Many have come here from not only the Northwest, but also from the Atlantic coast, at all points between Maryland and Maine, and found the change beneficial in catarrh, bronchial affections, and asthma.

There is one important item to be considered, which is, that all invalids whose cases demand a good deal of outdoor exercise, will be benefited here in winter, because it is seldom so cold as to keep a person in doors. Intense cold is utterly unknown, and probably always will be.

With an elevation of one thousand feet above the sea, in a region of high undulations, but not strictly mountainous, with no marshes, or wide river-bottoms, miasmatic diseases are seldom seen—never unless imported. Intermittents are no more seen in the hilly portions of East Tennessee than in New Hampshire or Vermont.

As I may have stated in some former communication, this region is well adapted to persons of middle or more advanced age, who have suffered from miasmatic diseases, and are left with various chronic ailments incident to them.

Phthisis pulmonalis is not prevalent here. It is seldom that we meet with a case of fatal lung disease which is not a direct result of acute pneumonia or pleurisy.

We find but little prevalent disease among children. Cholera infantum is but rarely seen. In white families, where an intelligent regard is paid to hygiene, the children grow up fat and stout. Scarlet fever, in a mild form, has occurred but once since 1867. The other diseases incident to childhood are commonly mild in type. In fine, it is seldom that any disease prevails epidemically. Chronic affections furnish a great proportion of the practice.

Uterine diseases, both functional and organic, are very common, especially among the class who either cannot, or will not, properly protect the feet from cold and dampness in winter and spring. There is, perhaps, in all, a want of firmness in the tissues, which render the people liable to some forms of disease, more than in the North.

In summer, the heat is continuous, but not on an average greater than in the Northern States. Sunstroke is almost unknown among these hills and mountains. I have heard of but one case in Knoxville which was called sunstroke, and that was doubtful in its character. There is probably no region east of the Rocky Mountains where the summer months can be spent with more comfort than among these hills. There are numerous watering-places all along the valleys, where people can stay for three or four months in summer with great benefit to the health. The mineral springs are found gushing out from some mountain or hillside, in a shaded and elevated spot, and generally much higher than this city. Great numbers come every summer from the Gulf States and spend the time very pleasantly, besides escaping from an unhealthy locality. This is, then, a happy medium between North and South, and a safe retreat from the regions of cold winter, on the one hand, and extremely hot summer, on the other.

I desire further to allude to the advantages afforded here to those, who, having spent the winter months farther South, find it necessary to leave on account of the heat, but do not wish to return to their homes till June. From the middle of March to the first of June, the weather here is delightful, and no pleasanter spot could be selected as a half-way point. The scenery is beautiful at all seasons; but in spring it is particularly so.

The above remarks are made through the columns of THE MEDICAL EXAMINER as a medium of communication to physicians who are desirous of obtaining information for the benefit of their patients and friends.

March 9th, 1874.

